# Cerebrotendinous Xanthomatosis: Report of Two Siblings With the Same Mutation but Variable Presentation

**DOI:** 10.7759/cureus.33378

**Published:** 2023-01-05

**Authors:** Nivedhan Mahadevan, Varshini Thiruvadi, Paranthakan C, Rekha A, Magesh A

**Affiliations:** 1 Internal Medicine, Thanjavur Medical College and Hospital, Thanjavur, IND; 2 Medical Genetics, Christian Medical College Vellore, Vellore, IND

**Keywords:** ctx, xanthoma, lipid metabolism, cyp27a1, cholestanol

## Abstract

Cerebrotendinous xanthomatosis (CTX), also known as CTX, is an extremely rare bile acid metabolic disorder caused by mutations in the cytochrome P450 family 27 subfamily A member 1 (CYP27A1) gene. This genetic disease is inherited in an autosomal recessive manner, and it affects the enzyme sterol 27-hydroxylase, which is involved in the bile acid metabolic process. It is distinguished by diarrhoea in infancy, early juvenile cataract, tendon xanthomas in adolescence, and progressive neuropsychiatric dysfunction in adulthood. So far, India has reported eight genetically confirmed cases. We present two cases of CTX among siblings in a family. The elder sibling was initially diagnosed, and after reviewing his family history and performing a thorough clinical examination, we discovered a similar manifestation in his younger sibling. Genetic testing on the siblings revealed similar mutations at exon 2 of the CYP27A1 gene. If a pathogenic mutation is discovered in a family member, prenatal and preimplantation genetic testing, as well as childhood screening, are the options. These screening strategies will prevent the onset of neuropsychiatric manifestations and disability.

## Introduction

Cerebrotendinous xanthomatosis (CTX), formerly known as cerebral cholesterinosis, cerebrotendinous cholesterosis, and Van Bogaert-Scherer-Epstein syndrome, is an exceedingly rare bile acid metabolic condition resulting from mutations in the cytochrome P450 family 27 subfamily A member 1 (CYP27A1) gene. This hereditary condition is transmitted in an autosomal recessive way and affects the bile acid metabolic enzyme sterol 27-hydroxylase. To this point, there have been 400 cases documented all across the world [1]. This article discusses an unusual case involving two siblings in a family. This metabolic derangement leads to elevated cholesterol and cholestanol accumulation in many sites, resulting in diverse clinical manifestations including diarrhoea in infancy, early juvenile cataract, tendon xanthomas in adolescence, and gradual neurological decline in maturity. In 75% of cases, cataracts manifest within the first decade of life. In the second and third decades, xanthomas are found in numerous locations. The onset of pyramidal and cerebellar symptoms occurs between the ages of 20 and 30. Over 50% of people get dementia with a serious intellectual decline in their thirties, while some have cognitive impairment as early as childhood. Symptoms of neuropsychiatric disorders include alterations in behaviour, hallucinations, anxiety, aggression, depression, and suicide attempts [2]. A proband is diagnosed with CTX based on suggestive evidence and the finding of biallelic pathogenic variants in CYP27A1 via molecular genetic testing [2]. Long-term treatment with chenodeoxycholic acid normalises plasma and CSF cholestanol levels and enhances neurophysiology. Statins alone or in combination with chenodeoxycholic acid reduce cholestanol levels and ameliorate clinical symptoms [1,2].

## Case presentation

Case 1

**Figure 1:**
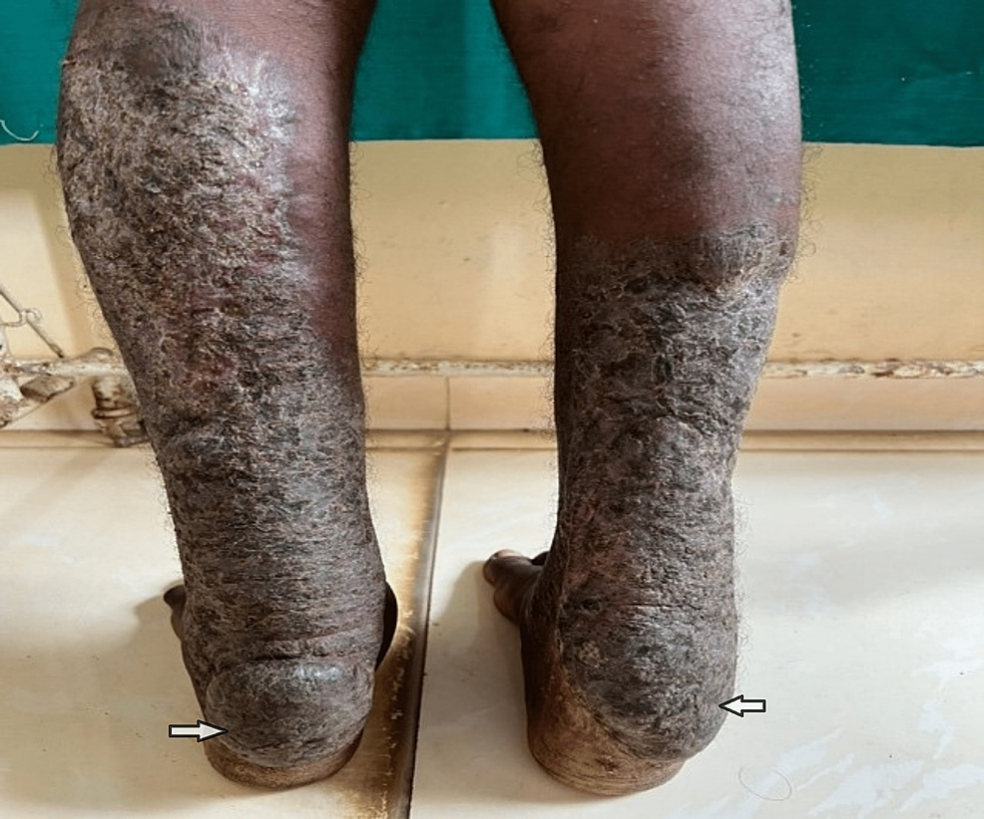
Bilateral achilles tendon xanthomas in case 1 (arrows)

**Figure 2:**
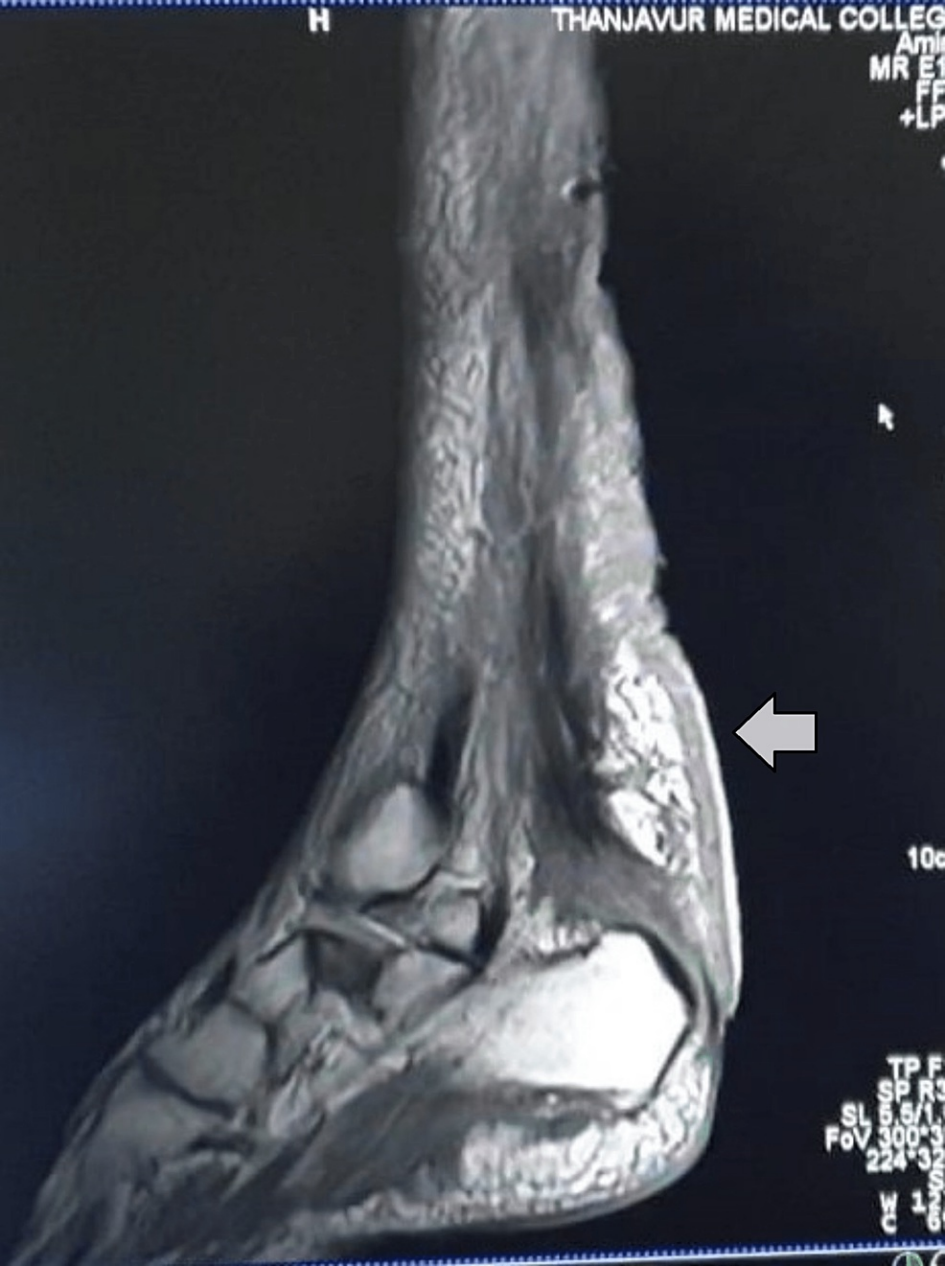
MRI of the right ankle in case 1 MRI T1 image in sagittal section showed tendon xanthoma with intermediate signal intensity in the tendo achilles (arrow)

**Figure 3:**
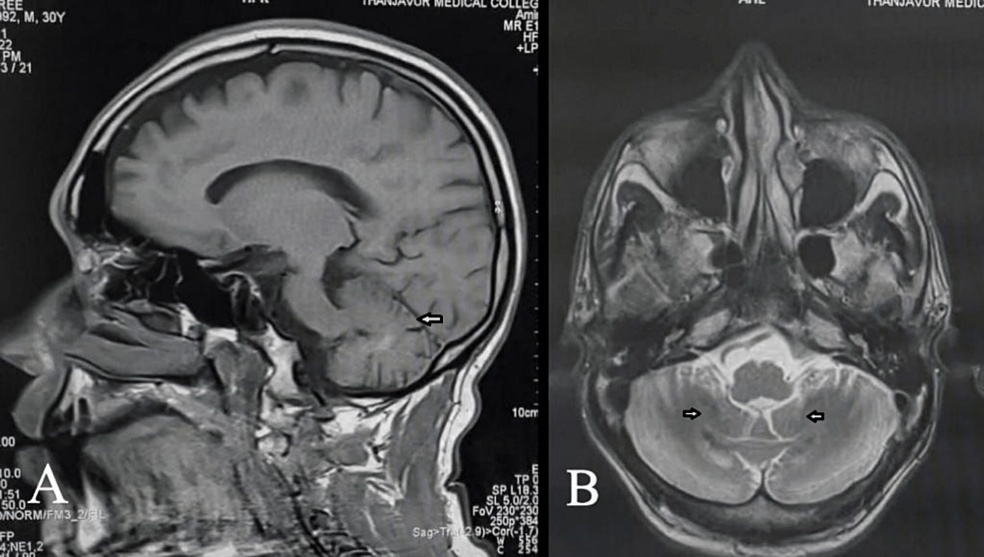
MRI brain finding of case 1 MRI brain revealed (A) T2 sagittal section showing cerebellar atrophy (arrow) and (B) T2 axial section showing bilateral symmetrical hypointensities involving dentate nuclei suggesting calcification (arrows)

**Figure 4:**
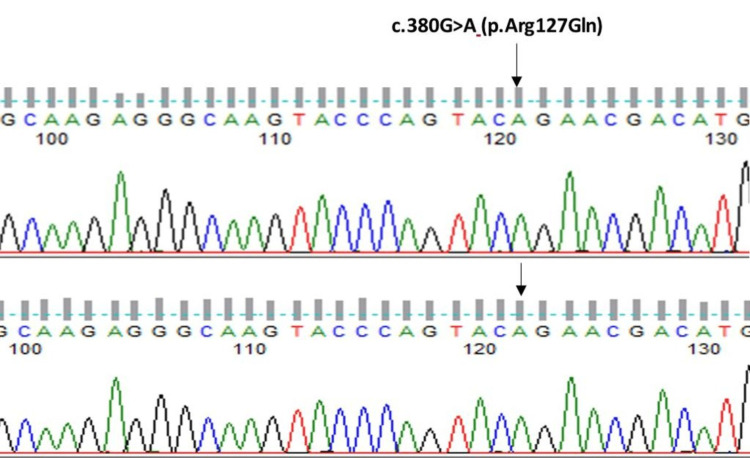
Electropherogram in affected siblings Electropherogram shows homozygous missense variant c.380G>A(p.Arg127Gln) denoted by arrows

A 30-year-old male, born out of a second-degree consanguineous marriage, presented with incoordination and difficulty walking straight since puberty, which progressively increased over the last year. History and clinical examination revealed bilateral tendon xanthomas on the Achilles and patellar tendons (Figure 1), an immature cataract in the right eye, pseudophakia in the left eye, a moderate intellectual disability, and cerebellar dysfunction in terms of ataxia and incoordination, which worsened recently. Laboratory investigations further revealed normal haematological parameters, a basic metabolic profile, and a normal lipid profile. Fine-needle aspiration cytology (FNAC) of the swelling from the achilles tendon revealed foamy histiocytes in clusters and occasional inflammatory cells suggestive of fibroxanthoma. MRI of the right ankle in the sagittal T1 section showed tendon enlargement with tendon xanthoma showing intermediate signal intensity (Figure 2), and MRI of the brain in the T2 sagittal and axial sections showed bilateral dentate nucleus calcification and bilateral cerebellar atrophy (Figure 3A-3B). Based on the above clinical picture, cerebrotendinous xanthomatosis was suspected, and genetic testing identified a homozygous missense variant c.380G>A in exon 2 of the CYP27A1 gene (ENST00000258415.9) that results in the substitution of amino acid at codon position 127 (p.Arg127Gln) (Figure 4). This variant has been previously reported in patients affected by CTX and classified as pathogenic (RCV000056111.7), thus substantiating the diagnosis. This patient was treated with atorvastatin 80 mg once daily and clopidogrel 75 mg once daily, as chenodexycholic acid was unavailable in India.

Case 2

**Figure 5:**
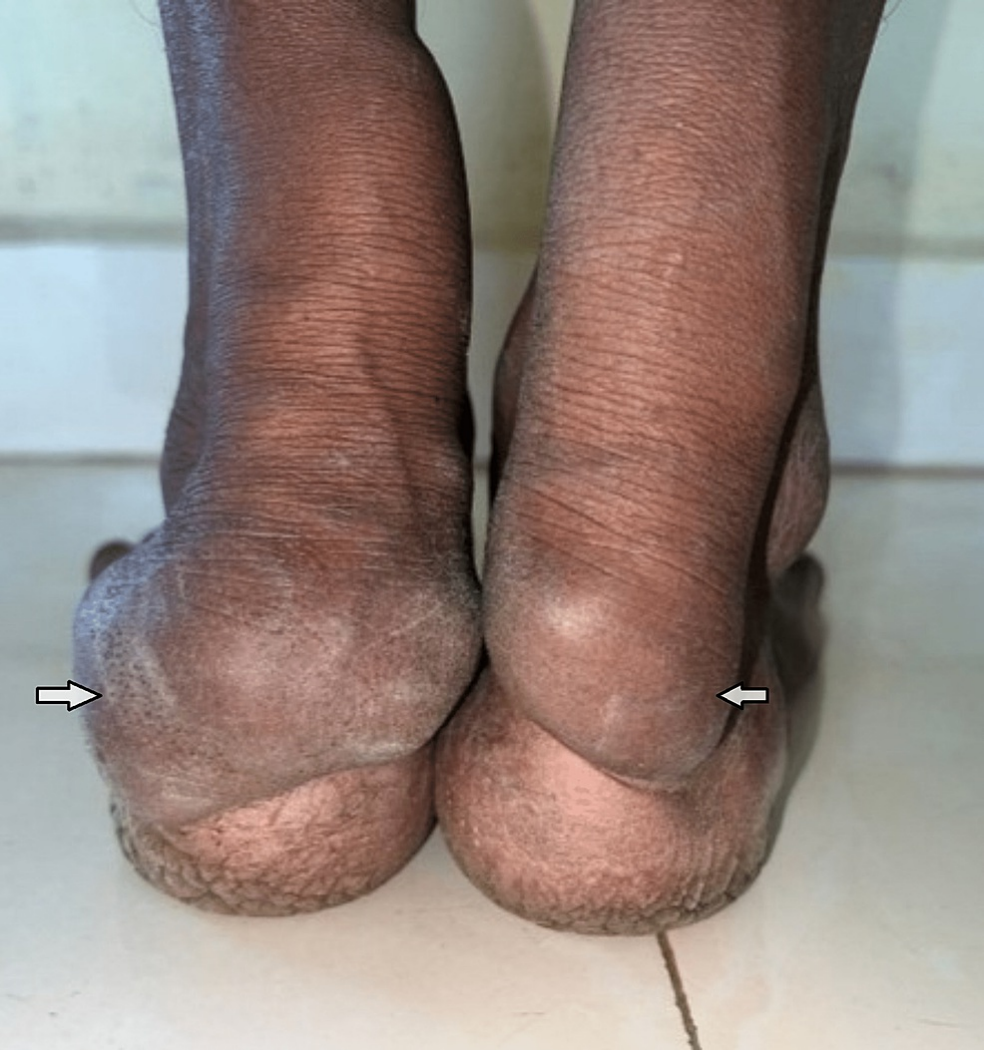
Bilateral achilles tendon xanthoma in case 2 (arrows)

**Figure 6:**
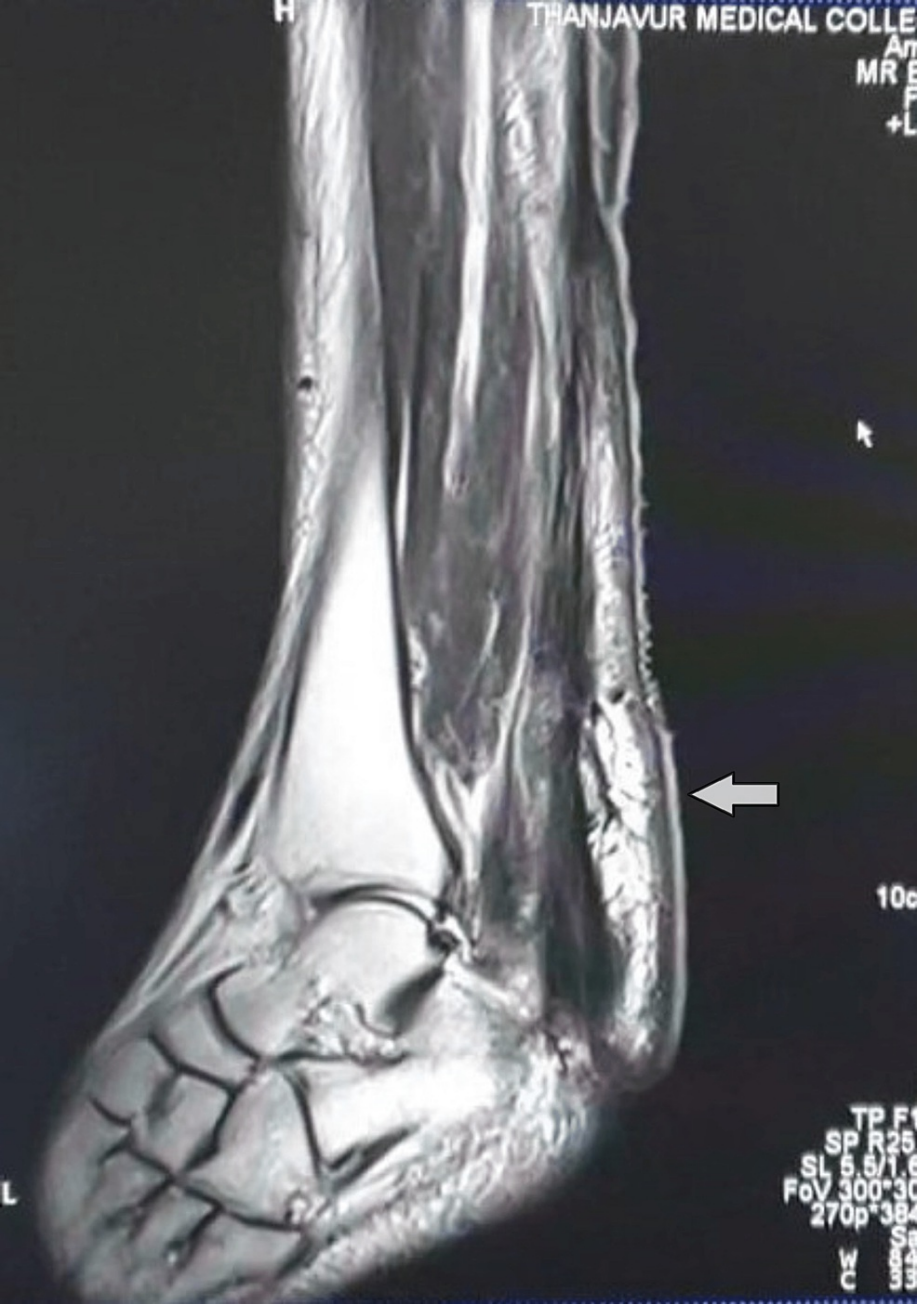
MRI image of the left ankle in case 2 MRI sagittal T1 image revealed tendon xanthoma in tendo achilles of left side (arrow)

**Figure 7:**
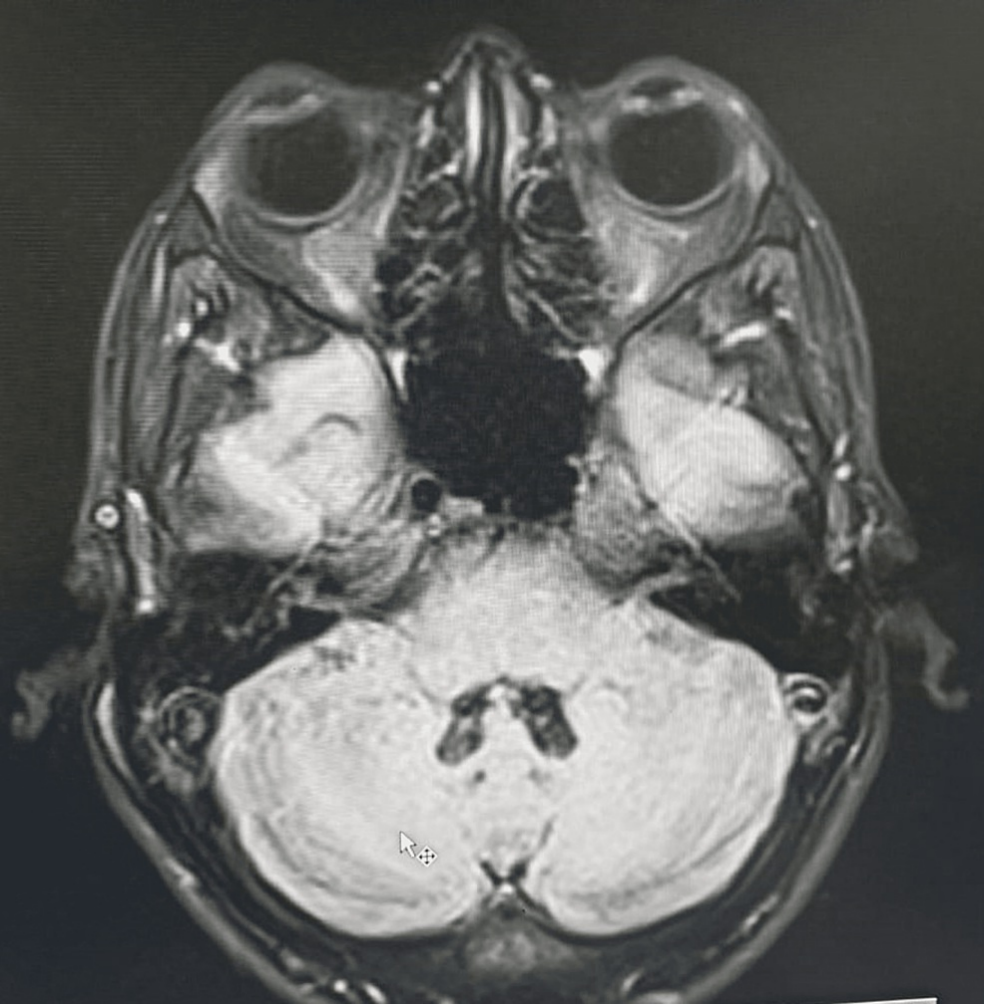
MRI brain findings in case 2 MRI brain FLAIR axial section showed normal bilateral dentate nucleus with no cerebellar atrophy (arrow)

A 27-year-old male, who is the younger sibling of the above-mentioned case, presented with bilateral Achilles (Figure 5), patellar tendon xanthomas since childhood, and a clinical neurological examination within normal limits. He also developed early-onset cataracts in both eyes, for which a posterior chamber intraocular lens (PCIOL) was placed. The psychiatric evaluation revealed a moderate intellectual disability. A FNAC of the swelling revealed features of fibroxanthoma. T1 sagittal MRI of the left ankle revealed tendon enlargement with tendon xanthoma showing intermediate signal intensity in the tendo Achilles (Figure 6), and FLAIR axial MRI of a younger sibling revealed normal bilateral dentate nucleus with no cerebellar atrophy (Figure 7). Laboratory investigations revealed a normal haematological and biochemical profile. Considering the above clinical presentation and the presence of a positive family history in his elder sibling, cerebrotendinous xanthomatosis was suspected, and the diagnosis was confirmed by genetic testing, which revealed the same homozygous missense pathogenic variant c.380G>A (p.Arg127Gln) in the CYP27A1 gene.

## Discussion

CTX is a rare condition initially documented in 1937; it is more common in females than males (55% vs. 45%). Three to five out of every 100,000 white Americans are estimated to have CTX, but Israelites and Jews of Moroccan ancestry have the highest incidence [3,4]. CTX is caused by mutations in the CYP27A1 gene on chromosome 2q33, which lead to the production of a suboptimal sterol 27-hydroxylase enzyme. This enzyme catalyses multiple steps in drug metabolism and the synthesis of lipids. This mitochondrial protein participates in the bile formation process by oxidising cholesterol intermediates and is also necessary for cholesterol homeostasis as the primary way of removing cholesterol from the body is by conversion of cholesterol to bile acids. 50% of all CYP27A1 mutations occur in exons 6-8, while 16% and 14% occur in exons 2 and 4, respectively. There are missense (about 45%), nonsense (20%), splice site (18%), deletion (14%), and insertion (2%) variants in all nine exons of CYP27A1 [4].

CTX progresses slowly and presents in numerous ways; the average age of diagnosis is 32 years. Clinical hallmarks include premature bilateral cataracts (88% of patients, based on a survey of selected case series from around the world), intractable diarrhoea (50%), progressive neurologic signs and symptoms (77% pyramidal; 62% cerebellar), and tendon xanthomas (68%). Only 4% (1/25 patients) experience osteoporosis, a saccular abdominal aortic aneurysm, or neonatal jaundice. Tendon xanthomas were most prevalent in the bilateral ankles, followed by the knees, elbow, fingers, and tibia. Bilateral cataracts, intellectual impairment, and recurrent diarrhoea are thought to be the early signs in children; however, these symptoms are frequently neglected until neurological abnormalities develop, resulting in a potentially fatal delay in diagnosis [5]. The actual cause of CTX-induced brain injury is unknown. A high quantity of cholestanol in brain tissue is considered to activate apoptotic pathways, resulting in neuronal death [6].

CTX can be diagnosed biochemically by detecting elevated blood levels of cholestanol or urine bile alcohol, as well as genetically by detecting mutations in the sterol-27-hydroxylase gene. While blood cholesterol levels are normal or reduced, cholestanol levels are often 5-10 times higher. Most people's plasma cholestanol levels are below 4.5 mg/L. CTX concentrations have been reported from 8.5 to 100.6 mg/L. Conventional MRI studies have revealed focal/diffuse white matter abnormalities as well as varying degrees of cerebral and cerebellar atrophy in CTX patients' brains [7]. Neonatal screening is a different approach to diagnosis that has been advocated as a solution for the early detection of CTX patients [8]. To obtain 100% test specificity 7α,12α-dihydroxy-4-cholesten-3-one (7α12αC4), 5β-cholestane-3α,7α,12α,25-tetrol glucuronide (GlcA-tetrol)/tauro-chenodeoxycholic acid (t-CDCA) ratio, and GlcA-tetrol are both effective CTX biomarkers for newborn screening [9,10]. Early-onset intracranial atherosclerosis is a rare form of presentation that may necessitate vigilant angiographic imaging techniques [11].

Statins are used in treatment, either alone or in combination with chenodeoxycholic acid [12]. Treatment with chenodeoxycholic acid at 750 mg/day in adults and 10-20 mg/kg/day in children normalises plasma and CSF levels of cholestanol and should be started as soon as possible to prevent irreversible damage to axons. This medication has also been shown to be effective in treating osteoporosis caused by CTX. The overall treatment approach is multidisciplinary, including cataract extraction, neuropsychiatric management, and tendon xanthoma excision.

## Conclusions

Cerebrotendinous xanthomatosis is a rare autosomal recessive disease that presents with tendon xanthomas, early-onset cataracts, and neuropsychiatric manifestations. Early diagnosis and timely treatment can attenuate the neuropsychiatric progression of the disease. Prenatal and preimplantation genetic testing and childhood screening are options if the CYP27A1 pathogenic mutations have been found in an affected family member. These screening strategies will aid in early treatment, thus preventing the development of neuropsychiatric manifestations and preventing disability.
